# Diisopropyl­ammonium 3,5,6-trichloro­pyridin-2-olate

**DOI:** 10.1107/S1600536808002389

**Published:** 2008-01-30

**Authors:** Zhi Yan Hu, Hui Zheng, Dan Qian Xu

**Affiliations:** a School of Science, Zhejiang Forestry University, Linan, Hangzhou 311300, People’s Republic of China; b State Key Laboratory Breeding Base of Green Chemistry-Synthesis Technology, Zhejiang University of Technology, Hangzhou 310014, People’s Republic of China

## Abstract

In the title salt, C_6_H_16_N^+^·C_5_HCl_3_NO^−^, the cation links to the anion, which is almost planar, through an N—H⋯O hydrogen bond. Inter­molecular hydrogen bonds link two cations and two anions into a centrosymmetric cluster. The atoms involved in the hydrogen bonding form a planar octa­gonal arrangement in the crystal structure.

## Related literature

For related literature, see: Fox *et al.* (2002 ▶); Baughman (1989 ▶); Fakhraian *et al.* (2004 ▶); Zheng, Liu, Li *et al.* (2006 ▶); Zheng, Liu, Xu *et al.* (2006*a* ▶,*b* ▶).
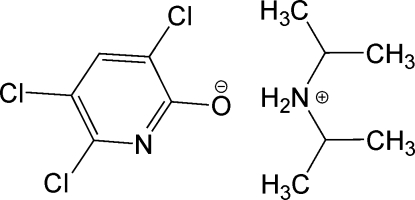


## Experimental

### 

#### Crystal data

C_6_H_16_N^+^·C_5_HCl_3_NO^−^
*M* *_r_* = 299.63
Monoclinic, 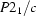

*a* = 8.087 (3) Å
*b* = 11.066 (3) Å
*c* = 16.389 (5) Å
β = 94.540 (15)°
*V* = 1462.1 (8) Å^3^
*Z* = 4
Mo *K*α radiation
μ = 0.61 mm^−1^
*T* = 298 (1) K
0.39 × 0.18 × 0.16 mm

#### Data collection

Rigaku R-AXIS RAPID diffractometer
Absorption correction: multi-scan (*ABSCOR*; Higashi, 1995 ▶) *T*_min_ = 0.794, *T*_max_ = 0.907
14096 measured reflections
3357 independent reflections
2081 reflections with *F* ^2^ > 2σ(*F* ^2^)
*R*_int_ = 0.027

#### Refinement

*R*[*F* ^2^ > 2σ(*F* ^2^)] = 0.035
*wR*(*F* ^2^) = 0.081
*S* = 1.04
3357 reflections
155 parameters
H-atom parameters constrained
Δρ_max_ = 0.29 e Å^−3^
Δρ_min_ = −0.31 e Å^−3^

### 

Data collection: *PROCESS-AUTO* (Rigaku, 1998 ▶); cell refinement: *PROCESS-AUTO*; data reduction: *CrystalStructure* (Rigaku/MSC, 2004 ▶); program(s) used to solve structure: *SIR97* (Altomare *et al.*, 1999 ▶); program(s) used to refine structure: *CRYSTALS* (Betteridge *et al.*, 2003 ▶); molecular graphics: *ORTEP-3 for Windows* (Farrugia, 1997 ▶); software used to prepare material for publication: *CrystalStructure*.

## Supplementary Material

Crystal structure: contains datablocks global, I. DOI: 10.1107/S1600536808002389/wk2076sup1.cif

Structure factors: contains datablocks I. DOI: 10.1107/S1600536808002389/wk2076Isup2.hkl

Additional supplementary materials:  crystallographic information; 3D view; checkCIF report

## Figures and Tables

**Table 1:** Hydrogen-bond geometry (Å, °)

*D*—H⋯*A*	*D*—H	H⋯*A*	*D*⋯*A*	*D*—H⋯*A*
N2—H201⋯O1	0.96	1.94	2.8803 (15)	166
N2—H201⋯N1	0.96	2.53	3.2556 (16)	133
N2—H202⋯O1^i^	0.96	1.85	2.7424 (16)	152

Symmetry code: (i) 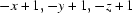.

**Fig. 1.**
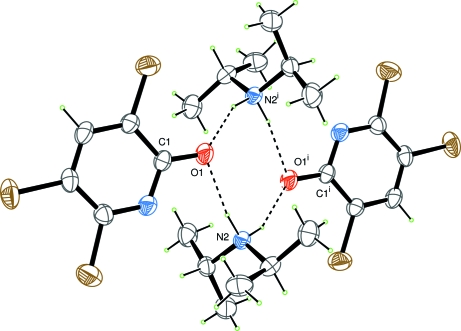
The centrosymmetric hydrogen-bonded (dashed lines) cluster in (I), showing the atom-numbering scheme and 40% probability displacement ellipsoids. [Symmetry code: (i) -x + 1, -y + 1, -z + 1.]

## supplementary crystallographic information

### Comment

Many compounds containing the 3,5,6-trichloro-pyridin-2-ol group have potential bioactivity (Fakhraian *et al.*, 2004; Baughman, 1989; Fox *et al.*, 2002). Similar compounds has been synthesized in our laboratory and some bioactive compounds have been found (Zheng *et al.*, 2006). In a continuation of our work into structure–activity relationships (Zheng, Liu, Xu & Xu, 2006*a*,b), we obtained a colorless crystalline compound, (I), by mixing sodium 3,5,6-trichloropyridin-2-olate with diisopropylammonium chloride, which was crystallized from diethyl ether. In the crystal structure, there are two independent structural units. The diisopropylammonium cation has an N2—C10 distance of 1.5009 (19)Å and a C10—N2—C7 angle of 117.47 (11)° (Table 1). The 3,5,6-trichloropyridin-2-olate anion has a C1—O1 distance of 1.2716 (18) Å, which is shorter than normal C—O distance for a smallar covalent radius with C*sp*^2^. The interesting feature of the crystal structure is the intermolecular hydrogen bonds N2—H201···O1 and N2—H202···O1^i^, which link two cations and two anions into a centrosymmetric cluster and form a planar octagon (Table 2 and Fig. 1).

### Experimental

Sodium 3,5,6-trichloropyridin-2-olate (2.2 g, 10 mmol) was dissolved in the distilled water (30 ml) at 370 K, cooled to room temperature, and diisopropylammonium chloride, which was generated from diisopropylamine (1.8 ml, 12 mmol) with HCl (36%) (2 ml), was added dropwise with stirring for 0.5 h. The solution was extracted with diethyl ether 2 × 15 ml. and dried over anhydrous magnesium sulfate. Suitable crystals (m.p. 442–443 K) were obtained from a diethyl ether solution.

### Refinement

All H atoms were placed in calculated positions, with C—H distances in the range 0.93–0.98 Å and N—H distance of 0.96 Å. All H atoms were refined using a riding model, with *U*_iso_(H)= 1.2*U*_eq_.

### Crystal data

**Table:**

C_6_H_16_N^+^·C_5_HCl_3_NO^–^	*F*_000_ = 624.00
*M**_r_* = 299.63	*D*_x_ = 1.361 Mg m^−^^3^
Monoclinic, *P*2_1_/*c*	Melting point: 443 K
Hall symbol: -P 2ybc	Mo *K*α radiation λ = 0.71075 Å
*a* = 8.087 (3) Å	Cell parameters from 10937 reflections
*b* = 11.066 (3) Å	θ = 3.1–27.5º
*c* = 16.389 (5) Å	µ = 0.61 mm^−^^1^
β = 94.540 (15)º	*T* = 298 (1) K
*V* = 1462.1 (8) Å^3^	Block, colorless
*Z* = 4	0.39 × 0.18 × 0.16 mm

### Data collection

**Table:**

Rigaku R-AXIS RAPID diffractometer	2081 reflections with *F*^2^ > 2σ(*F*^2^)
Detector resolution: 10.00 pixels mm^-1^	*R*_int_ = 0.027
ω scans	θ_max_ = 27.5º
Absorption correction: multi-scan(ABSCOR; Higashi, 1995)	*h* = −9→10
*T*_min_ = 0.794, *T*_max_ = 0.907	*k* = −14→14
14096 measured reflections	*l* = −21→21
3357 independent reflections	

### Refinement

**Table:**

Refinement on *F*^2^	*w* = 1/[0.0002*F*_o_^2^ + σ(*F*_o_^2^)]/(4*F*_o_^2^)
*R*[*F*^2^ > 2σ(*F*^2^)] = 0.035	(Δ/σ)_max_ < 0.001
*wR*(*F*^2^) = 0.081	Δρ_max_ = 0.29 e Å^−^^3^
*S* = 1.05	Δρ_min_ = −0.31 e Å^−^^3^
3357 reflections	Extinction correction: Larson (1970), equation 22
155 parameters	Extinction coefficient: 143 (15)
H-atom parameters constrained	

### Special details

**Table:**

Refinement. Refinement using all reflections. The weighted *R*-factor (*wR*) and goodness of fit (*S*) are based on *F*^2^. *R*-factor (gt) are based on *F*. The threshold expression of *F*^2^ > 2.0 σ(*F*^2^) is used only for calculating *R*-factor (gt).

### Fractional atomic coordinates and isotropic or equivalent isotropic displacement parameters (Å^2^)

**Table:**

	*x*	*y*	*z*	*U*_iso_*/*U*_eq_	
Cl1	0.39967 (6)	0.13293 (4)	0.40436 (3)	0.08518 (18)	
Cl2	0.05102 (6)	−0.02373 (5)	0.65045 (3)	0.08680 (17)	
Cl3	0.06254 (8)	0.24633 (5)	0.71793 (4)	0.1034 (2)	
O1	0.36820 (13)	0.36964 (9)	0.49068 (6)	0.0604 (3)	
N1	0.22849 (16)	0.29782 (11)	0.59411 (9)	0.0566 (4)	
N2	0.37423 (14)	0.57219 (10)	0.60126 (6)	0.0469 (3)	
C1	0.30288 (18)	0.28003 (12)	0.52456 (10)	0.0498 (4)	
C2	0.30287 (19)	0.16000 (13)	0.49307 (10)	0.0529 (4)	
C3	0.2269 (2)	0.06825 (13)	0.53135 (11)	0.0595 (5)	
C4	0.1497 (2)	0.09140 (13)	0.60194 (10)	0.0573 (4)	
C5	0.1557 (2)	0.20776 (14)	0.62985 (10)	0.0573 (5)	
C6	0.5796 (2)	0.46023 (17)	0.68652 (12)	0.0771 (6)	
C7	0.4207 (2)	0.53190 (13)	0.68790 (9)	0.0561 (4)	
C8	0.4384 (2)	0.63777 (17)	0.74638 (11)	0.0780 (6)	
C9	0.0708 (2)	0.58893 (18)	0.60955 (12)	0.0764 (6)	
C10	0.2255 (2)	0.65272 (13)	0.58681 (10)	0.0570 (4)	
C11	0.2132 (2)	0.69139 (17)	0.49810 (11)	0.0742 (6)	
H3	0.2274	−0.0097	0.5101	0.071*	
H7	0.3333	0.4784	0.7051	0.067*	
H10	0.2433	0.7245	0.6214	0.068*	
H61	0.6635	0.5111	0.6664	0.091*	
H62	0.5625	0.3911	0.6515	0.092*	
H63	0.6146	0.4340	0.7410	0.092*	
H81	0.3347	0.6800	0.7466	0.093*	
H82	0.4713	0.6094	0.8006	0.093*	
H83	0.5216	0.6914	0.7286	0.093*	
H91	−0.0234	0.6409	0.5988	0.093*	
H92	0.0557	0.5167	0.5774	0.092*	
H93	0.0819	0.5681	0.6666	0.093*	
H111	0.1926	0.6214	0.4642	0.088*	
H112	0.3152	0.7287	0.4854	0.088*	
H113	0.1237	0.7479	0.4883	0.088*	
H201	0.3549	0.5015	0.5680	0.056*	
H202	0.4689	0.6125	0.5820	0.057*	

### Atomic displacement parameters (Å^2^)

**Table:**

	*U*^11^	*U*^22^	*U*^33^	*U*^12^	*U*^13^	*U*^23^
Cl1	0.1051 (4)	0.0724 (3)	0.0820 (3)	−0.0167 (2)	0.0323 (2)	−0.0223 (2)
Cl2	0.1076 (4)	0.0690 (3)	0.0828 (3)	−0.0353 (2)	0.0014 (2)	0.0219 (2)
Cl3	0.1362 (5)	0.0953 (4)	0.0860 (4)	−0.0262 (3)	0.0534 (3)	−0.0126 (2)
O1	0.0653 (7)	0.0426 (5)	0.0749 (7)	−0.0111 (5)	0.0165 (5)	0.0024 (5)
N1	0.0615 (8)	0.0436 (7)	0.0654 (9)	−0.0082 (6)	0.0109 (6)	−0.0044 (6)
N2	0.0512 (7)	0.0390 (6)	0.0509 (7)	−0.0053 (5)	0.0052 (5)	−0.0037 (5)
C1	0.0464 (8)	0.0419 (8)	0.0603 (10)	−0.0055 (6)	−0.0003 (7)	0.0006 (7)
C2	0.0559 (9)	0.0458 (8)	0.0570 (9)	−0.0060 (7)	0.0036 (7)	−0.0039 (7)
C3	0.0691 (10)	0.0399 (8)	0.0675 (11)	−0.0095 (7)	−0.0064 (8)	−0.0031 (7)
C4	0.0622 (10)	0.0498 (9)	0.0582 (10)	−0.0147 (7)	−0.0059 (8)	0.0108 (8)
C5	0.0611 (10)	0.0559 (9)	0.0552 (10)	−0.0084 (8)	0.0063 (8)	0.0022 (7)
C6	0.0878 (13)	0.0710 (11)	0.0703 (12)	0.0083 (10)	−0.0086 (10)	0.0142 (10)
C7	0.0666 (10)	0.0507 (8)	0.0511 (9)	−0.0089 (7)	0.0052 (7)	0.0077 (7)
C8	0.1041 (15)	0.0719 (12)	0.0562 (10)	−0.0103 (10)	−0.0058 (10)	−0.0070 (9)
C9	0.0577 (11)	0.0899 (13)	0.0834 (13)	0.0033 (9)	0.0158 (9)	−0.0142 (11)
C10	0.0581 (9)	0.0469 (8)	0.0652 (10)	0.0045 (7)	−0.0003 (8)	−0.0107 (7)
C11	0.0690 (12)	0.0679 (11)	0.0833 (13)	0.0045 (9)	−0.0092 (10)	0.0105 (10)

### Geometric parameters (Å, °)

**Table:**

Cl1—C2	1.7310 (17)	C6—H62	0.960
Cl2—C4	1.7297 (17)	C6—H63	0.960
Cl3—C5	1.7337 (18)	C7—C8	1.513 (2)
O1—C1	1.2716 (18)	C7—H7	0.980
N1—C1	1.345 (2)	C8—H81	0.960
N1—C5	1.318 (2)	C8—H82	0.960
N2—C7	1.5072 (18)	C8—H83	0.960
N2—C10	1.5009 (19)	C9—C10	1.508 (2)
N2—H201	0.959	C9—H91	0.960
N2—H202	0.961	C9—H92	0.960
C1—C2	1.425 (2)	C9—H93	0.960
C2—C3	1.365 (2)	C10—C11	1.511 (2)
C3—C4	1.382 (2)	C10—H10	0.980
C3—H3	0.930	C11—H111	0.960
C4—C5	1.366 (2)	C11—H112	0.960
C6—C7	1.512 (2)	C11—H113	0.960
C6—H61	0.960		
			
C1—N1—C5	120.76 (13)	N2—C7—C8	111.82 (12)
C7—N2—C10	117.47 (11)	N2—C7—H7	108.5
C7—N2—H201	108.2	C6—C7—C8	112.20 (14)
C7—N2—H202	107.5	C6—C7—H7	108.7
C10—N2—H201	107.6	C8—C7—H7	108.6
C10—N2—H202	108.8	C7—C8—H81	109.9
H201—N2—H202	106.8	C7—C8—H82	109.8
O1—C1—N1	119.02 (13)	C7—C8—H83	108.7
O1—C1—C2	123.84 (14)	H81—C8—H82	109.5
N1—C1—C2	117.14 (13)	H81—C8—H83	109.5
Cl1—C2—C1	118.58 (12)	H82—C8—H83	109.5
Cl1—C2—C3	120.48 (12)	C10—C9—H91	109.7
C1—C2—C3	120.93 (15)	C10—C9—H92	109.0
C2—C3—C4	119.79 (14)	C10—C9—H93	109.7
C2—C3—H3	120.1	H91—C9—H92	109.5
C4—C3—H3	120.1	H91—C9—H93	109.5
Cl2—C4—C3	120.26 (12)	H92—C9—H93	109.5
Cl2—C4—C5	123.09 (13)	N2—C10—C9	110.68 (12)
C3—C4—C5	116.66 (14)	N2—C10—C11	108.09 (13)
Cl3—C5—N1	115.00 (12)	N2—C10—H10	108.1
Cl3—C5—C4	120.30 (13)	C9—C10—C11	112.20 (14)
N1—C5—C4	124.70 (15)	C9—C10—H10	108.9
C7—C6—H61	108.8	C11—C10—H10	108.8
C7—C6—H62	110.2	C10—C11—H111	108.9
C7—C6—H63	109.4	C10—C11—H112	109.8
H61—C6—H62	109.5	C10—C11—H113	109.7
H61—C6—H63	109.5	H111—C11—H112	109.5
H62—C6—H63	109.5	H111—C11—H113	109.5
N2—C7—C6	106.86 (13)	H112—C11—H113	109.5
			
C1—N1—C5—Cl3	−178.90 (11)	N1—C1—C2—Cl1	−179.19 (11)
C1—N1—C5—C4	0.7 (2)	N1—C1—C2—C3	1.5 (2)
C5—N1—C1—O1	178.24 (13)	Cl1—C2—C3—C4	−179.77 (12)
C5—N1—C1—C2	−1.6 (2)	C1—C2—C3—C4	−0.4 (2)
C7—N2—C10—C9	−62.42 (16)	C2—C3—C4—Cl2	179.30 (12)
C7—N2—C10—C11	174.34 (12)	C2—C3—C4—C5	−0.5 (2)
C10—N2—C7—C6	−176.04 (12)	Cl2—C4—C5—Cl3	0.2 (2)
C10—N2—C7—C8	−52.90 (18)	Cl2—C4—C5—N1	−179.41 (12)
O1—C1—C2—Cl1	1.0 (2)	C3—C4—C5—Cl3	179.98 (9)
O1—C1—C2—C3	−178.36 (14)	C3—C4—C5—N1	0.4 (2)

### Hydrogen-bond geometry (Å, °)

**Table:**

*D*—H···*A*	*D*—H	H···*A*	*D*···*A*	*D*—H···*A*
N2—H201···O1	0.96	1.94	2.8803 (15)	166
N2—H201···N1	0.96	2.53	3.2556 (16)	133
N2—H202···O1^i^	0.96	1.86	2.7424 (16)	152

Symmetry codes: (i) −*x*+1, −*y*+1, −*z*+1.
